# The dataset of scanning electron microscope images of silver nanoparticles formed in situ by dopamine chemistry

**DOI:** 10.1016/j.dib.2018.08.172

**Published:** 2018-09-01

**Authors:** Tzu-Lan Chang, Tianchi Liu, Jun F. Liang

**Affiliations:** Stevens Institute of Technology, United States

## Abstract

The mussel inspired chemistry of dopamine oxidation to form polydopamine (PDA) and in situ reduction of metal ions in solution to form metal nanoparticles have widely opened the application of metal nanoparticles surface modification technology. This article contains the dataset of the scanning electron microscope (SEM) images of silver nanoparticles coated on polyethylene terephthalate (PET) films utilizing dopamine chemistry alone or combined with polyvinylpyrrolidone or glucose. The Ag NPs formed in various environments present round, cubic, or triangle shape. Mendeley Data, http://dx.doi.org/10.17632/bjjrt2dwbn.1.

**Specifications Table**

**Table:** 

Subject area	*Surface Chemistry*
More specific subject area	*Metal nanoparticles surface modification*
Type of data	*Image*
How data was acquired	*Scanning Electron Microscope (Auriga Modular Cross Beam workstation, Carl Zeiss, Inc.)*
Data format	*Raw*
Experimental factors	*PET films were immersed in alkaline dopamine solution for 0.5–24* *h before coating with Ag nanoparticles.*
Experimental features	*Polydopamine coated film in combination with or without reducing reagents will reduce silver ions into silver metals and the silver metals will aggregate to form silver nanoparticles of various shapes.*
Data source location	*Stevens Institute of Technology, Hoboken, NJ, USA*
Data accessibility	*Mendeley Data.*http://dx.doi.org/10.17632/bjjrt2dwbn.1

**Value of the data**

• The SEM images clearly show the various shapes of the Ag nanoparticles on the surfaces of the polyethylene terephthalate. The dopamine chemistry is universal and it could apply to various materials.
• The shape of the Ag nanoparticles will be different by utilizing different experimental parameters.
• The SEM images serve as a good guide to surface modification utilizing dopamine chemistry and Ag nanoparticles.

## 1 Data

The data contain SEM images of Ag nanoparticles coated on the surface of polyethylene terephthalate films. The Ag nanoparticles were formed utilizing dopamine chemistry alone or combined with polyvinylpyrrolidone or glucose.

## 2 Experimental design, materials and methods

### 2.1 Materials

The commercial polyethylene terephthalate (PET) membranes were purchased from GoodFellow Cambridge Limited (thickness: 0.023 mm). The PET film was cut into 1 in.^2^, shaken in absolute ethanol three times to clean the film, and followed by soaking in absolute ethanol for at least 18 h. The PET film was air-dried prior to use. Dopamine hydrochloride, silver nitrate, polyvinylpyrrolidone, glucose, and sodium hydroxide were purchased from Sigma-Aldrich (St. Louis, MO, USA). All the solutions were prepared with Milli-Q purified water (Millipore, ≥18.2 MΩ).

### 2.2 in situ formation of PDA-coated surfaces

Dopamine was dissolved in a 7:3 mixture of methanol and water [1] at a concentration of 2.04 mg/mL. 400 µL of 0.1 M NaOH solution was added to 19.6 mL of the as-prepared dopamine solution to make the as-prepared alkaline dopamine solution at a final concentration of 2.00 mg/mL [2]. The PET film (1 in.^2^) was immersed to the dopamine solution immediately. This will allow self-polymerization of dopamine under alkaline condition and the polydopamine (PDA) formed will coat on both sides of the film. The solutions were kept for 0.5 to 24 h at room temperature to prepare films with varying PDA thickness. The film was rinsed in 10 mL of water for 10 min in a bath-type sonicator to remove unbound dopamine monomer, dipped in absolute ethanol, air dried and kept in the dark for further modification.

### 2.3 in situ formation of Ag nanoparticles on PDA-coated surfaces

The following reactions took place in the dark at room temperature. The as-prepared films were rinsed with water to remove unbound silver ions. The films were dipped in absolute ethanol, air-dried, and kept in the dark for characterization.

#### 2.3.1 Silver ion auto-reduction on PDA-coated surfaces by PDA alone

The 0.5–24 h PDA-coated films (1 in.^2^) were immersed into 20 mL of 50–400 mM of silver nitrate solution for 18–72 h.

#### 2.3.2 Silver ion auto-reduction on PDA-coated surfaces by PDA and polyvinylpyrrolidone (PVP)

The 6–24 h PDA-coated films were immersed into 20 mL of 100 mM of polyvinylpyrrolidone solution in 50–400 mM of silver nitrate solution for 18 h.

#### 2.3.3 Silver ion auto-reduction on PDA-coated surfaces by PDA and followed by adding glucose

The 6 h PDA-coated film was immersed into 20 mL of 50 mM of silver nitrate solution for 18 h, and 0.1–10.0 mM glucose was then added into the silver nitrate solution and auto-reduction happened for another 24 h.

### 2.4 Study the morphologies of the Ag nanoparticles using scanning electron microscope (SEM)

Auriga Modular Cross Beam workstation (Carl Zeiss, Inc.) was using with 1.5 kV accelerating voltage. The films were sputter coated with a conductive layer of gold palladium (Au/Pd) for 1.0 min for an approximately 15 nm coating [3].
