# Supplementary material for: The dataset of scanning electron microscope images of silver nanoparticles formed in situ by dopamine chemistry
Source: Data Brief. 2018 Sep 1;20:1090–2. doi: 10.1016/j.dib.2018.08.172 (PMC6139999; doi:10.1016/j.dib.2018.08.172)
Supplement: Supplementary file 1 — Supplementary material [file mmc1.docx]

Manuscript No.: DIB-D-17-00812

Title: The dataset of scanning electron microscope images of silver nanoparticles formed in situ by dopamine chemistry

Journal Title: Data in Brief Corresponding

Corresponding Author: Miss Tzu-Lan Chang

Conflicts of interest: none
